# Dual Heterozygous Mutations in *CYP21A2* and *CYP11B1* in a Case of Nonclassic Congenital Adrenal Hyperplasia

**DOI:** 10.1016/j.aace.2022.10.003

**Published:** 2022-10-21

**Authors:** Eric D. Frontera, Joshua J. Brown, Hagop Ghareebian, Cary Mariash

**Affiliations:** 1 Department of Medicine, Indiana University School of Medicine, Indianapolis, Indiana; 2 Division of Endocrinology and Metabolism, Indiana University School of Medicine, Indianapolis, Indiana

**Keywords:** nonclassic, congenital, adrenal, hyperplasia, mutations, CAH, congenital adrenal hyperplasia, COVID-19, Coronavirus disease 2019, CYP, cytochrome, Gly, glycine, HSD, 3-beta-hydroxysteroid dehydrogenase, Leu, Leucine, NCCAH, nonclassic congenital adrenal hyperplasia, PCOS, polycystic ovarian syndrome, Phenylalanine, phenylanine, POR, P450 oxioreductase, STAR, steroidogenic acute regulatory protein gene, Val, valine, VUS, variant of undetermined significance

## Abstract

**Background/Objective:**

Nonclassic congenital adrenal hyperplasia (NCCAH) may be overlooked or mistaken for polycystic ovarian syndrome. Unlike congenital adrenal hyperplasia (CAH), the enzymatic activities of 21-hydroxylase or 11β-hydroxylase in NCCAH are not completely lost. In this case, NCCAH presented in a patient with *CYP21A2* and *CYP11B1* heterozygous mutations, one of which is a variant of unknown significance in *CYP11B1*.

**Case Report:**

A 30-year-old woman presented with a chief complaint of irregular menses and hirsutism. Previous medical history was significant for a prolactin level of 34.7 ng/mL (reference range, 2.0-23.0 ng/mL), a total serum testosterone level of 77 ng/dL (reference range, 25-125 ng/dL, not sex-specific), and a 2-mm × 3-mm pituitary lesion. An adrenocorticotrophic hormone stimulation test increased the 17-hydroxyprogesterone level from 444 ng/dL at baseline to 837 ng/dL at 60 minutes (baseline female reference range and stimulated reference ranges are 10-300 ng/dL and <1000 ng/dL, respectively). Gene sequencing revealed a heterozygous pathogenic *CYP21A2* variant and a heterozygous, previously undescribed variant of unknown significance in *CYP11B1*.

**Discussion:**

Unlike CAH, NCCAH presents more subtly and later in life, and salt wasting and hypertension are not typically seen. Although mutations in *CYP11B1* that cause steroid 11β-hydroxylase deficiency more commonly lead to the CAH phenotype, cases have been reported of *CYP11B1* mutations leading to NCCAH, depending on the location of the mutations.

**Conclusion:**

This patient’s case demonstrates physical examination and laboratory findings suggestive of NCCAH. Our case adds to the database of described mutations in *CYP11B1* and suggests that heterozygous mutations in 2 different genes may present phenotypically as NCCAH.

**Highlights**

• Nonclassic congenital adrenal hyperplasia (NCCAH) may go undiagnosed in women.
• The number of known mutations in *CYP11B1* causing CAH and nonclassic CAH is growing
• A digenic cause with heterozygous variants may be a genetic makeup of the condition

**Clinical Relevance**

The genetic diversity of nonclassic congenital adrenal hyperplasia ought to remind clinicians to consider it as a diagnosis, particularly in young women whose hyperandrogenism or menstrual irregularities cannot be explained by polycystic ovarian syndrome or another presumed endocrine abnormality when the patient is not responding to standard medical treatment.

## Introduction

Congenital adrenal hyperplasia (CAH) is a condition caused by various autosomal recessive mutations that lead to enzymatic dysfunction in steroidogenesis. It is classically caused by a defective steroid 21-hydroxylase enzyme from a mutation that produces a classic constellation of symptoms in the neonatal period, including genital virilization, hyponatremia, hyperkalemia, and adrenal insufficiency or crisis.^1^ A more common but often overlooked constellation of symptoms can occur, known as nonclassic congenital adrenal hyperplasia (NCCAH), which often presents later in childhood, adolescence, or adulthood. It is characterized by precocious puberty, menstrual irregularity, hirsutism, and acne and can be mistaken for polycystic ovarian syndrome (PCOS).^2^

A less common cause of CAH is a defect in steroid 11β-hydroxylase from a mutation in *CYP11B1*, resulting in hypertension, hypokalemia (in contrast to the salt-wasting symptoms of classic steroid 21-hydroxylase deficiency), and androgen excess symptoms.^3^ The severity of clinical symptoms is determined by the degree to which the mutation impairs the function of the affected enzyme.^4^ In rare cases, it is possible for an individual to have 2 separate mutations in both 21-hydroxylase and 11β-hydroxylase.^5^, ^6^, ^7^, ^8^, ^9^, ^10^ There is a paucity of reports of such dual mutations in the literature, and the clinical manifestations of such a case are not thoroughly described. Here we report a case wherein a pair of heterozygous mutations in both *CYP2A12* and *CYP11B1* was detected in a new diagnosis of NCCAH. The mutation detected in *CYP11B1* is a previously undescribed mutation.

## Case Report

The patient is a 30-year-old woman who presented with a chief complaint of irregular menses and hirsutism. Since adolescence, she menstruated every 2 to 3 months, accompanied by mild cramping. Her menstrual flow typically lasted for 6 to 7 days, and she occasionally had spotting between periods. Her facial hair was bothersome enough to require regular shaving. She never had acne, breast tenderness, or breast discharge. She experienced headaches once or twice a month but denied experiencing blurry vision and diplopia. She had never had children or tried to become pregnant but wanted to start a family with her husband in the coming years. She denied experiencing changes in thirst or urination, muscle aches, skin changes, bruising, rashes, frequent infections, anxiety, or depression. She denied the use of tobacco, alcohol, or recreational substances. She had a family history of irregular menses (her mother and sister) and type 1 diabetes (her mother and grandmother). Physical examination revealed no visual field defects, facial plethora, or telangiectasia. There was hirsutism at the chin. There was no goiter. Her skin was warm and dry, with no obvious rashes or bruises, and her abdomen was soft, with normal hair distribution and typical external genitalia for her age. Her body mass index was 23, her heart rate was 86 beats per minute, and her blood pressure was 124/80. Her previous medical history included a prolactin level of 34.7 ng/mL (reference range, 2.0-23.0 ng/mL) and an am total serum testosterone level of 77 ng/dL (reference range, 25-125 ng/dL, not sex-specific). She also had a 2-mm × 3-mm posterior pituitary lesion on magnetic resonance imaging. A pelvic ultrasound 3 years before presentation was reported to be normal. Other laboratory parameters before the initial evaluation can be found in Table 1. As part of this medical team’s initial evaluation, laboratory findings revealed the following: the am 17-hydroxyprogesterone level was 554 ng/dL (female reference range, 10-300 ng/dL), the total testosterone measured by Liquid Chromatography-Tandem Mass Spectrometry level was 83 ng/dL (reference range for females and children, 9-55 ng/dL), and the prolactin level was 29.6 ng/mL (reference range, 2.0-23.0 ng/mL). The findings of the further laboratory testing performed at that time can be found in Table 2. The patient was started on cabergoline (0.25 mg) biweekly, which decreased her prolactin level to 4.4 ng/dL in 4 weeks. At the lowered prolactin level, the patient continued to report menstrual irregularities and hirsutism. A 250-mcg cosyntropin test was performed. The am 17-hydroxyprogesterone level increased from 44 ng/dL at time 0 to 837 ng/dL at 60 minutes (Table 3). The adrenocorticotrophic hormone level was 178 pg/mL (reference range, 7.2-63 pg/mL) and the baseline am cortisol level was 24.1 mcg/dL (reference range, 8-25 mcg/dL). After a 1-mg dexamethasone suppression test, her serum am dexamethasone level was 197 ng/dL (140-295 ng/dL for 1-mg dexamethasone ingested the previous evening) and her am serum cortisol level was 0.5 mcg/dL (8-24 mcg/dL) (Table 3).

**Table 1:** Initial Laboratory Work-up

Test	Result	Reference range
Na (mmol/L)	138	135-145
K (mmol/L)	4.4	3.5-5.0
Cl (mmol/L)	107	98-108
HCO_3_ (mmol/L)	21	22-29
BUN (mg/dL)	10	5-20
Creatinine (md/dL)	0.74	0.60-1.20
Glucose (mg/dL)	102	70-99
Ca (mg/dL)	9	8.5-10.5
Alkaline phosphatase (units/L)	51	25-125
AST (units/L)	31	13-39
ALT (units/L)	10	7-52
Total bilirubin (mg/dL)	0.5	0.0-1.0
Total protein (g/dL)	6.8	6.4-8.0
Albumin (g/dL)	4.2	2.5-5.0
TSH (mcU/nL)	2.086	0.400-4.200
**Prolactin (ng/mL)**	**34.7**	**2.0-23.0**
**Total testosterone serum(ng/dL)**	**77**	**25-125**

Abbreviations: Na = sodium; K= potassium; Cl = chloride; HCO_3_= Bicarbonate; BUN = Blood Urea Nitrogen; Ca = Calcium; AST = Aspartate Transaminase; ALT = Alanine Transaminase; TSH = Thyroid Stimulating Hormone

Abnormal baseline Prolactin level bolded.

Total testosterone level bolded in this female patient.

**Table 2:** Endocrine Work-up for Hyperandrogenism

Test	Result	Reference range
**17-OH progesterone (ng/dL)**	**554**	**10-300 ng/dL**
**Total testosterone F/C LC-MS/MS(ng/dL)**	**83**	**9-55**
**Serum prolactin (ng/mL)**	**29.6**	**2.0-23.0**
DHEA sulfate (mcg/dL)	223	35-430
Estradiol (pg/mL)	261	N/A
IGF-1 (ng/mL)	202	87-368
Serum sex hormone–binding globulin (nmol/L)	106.61	30-135
Free testosterone F/C LC-MS/MS (pg/mL)	6.6	0.8-7.4
FSH (mU/mL)	11.5	N/A
LH (mU/mL)	35.5	N/A

Abbreviations: OH = hydroxy; F/C = Females and Children; LC-MS/MS = Liquid Chromatography-Tandem Mass Spectrometry; DHEA = Dehydroepiandrosterone; IGF-1 = Insulin-like Growth Factor 1; FSH = Follicular Stimulating Hormone; LH = Leutinizing Hormone; N/A = Not Available (due to known phase of ovulation)

Abnormal labs bolded.

**Table 3:** Adrenocorticotropic Hormone Stiumation Test, Dexamethasone Suppression Test, and Repeat FSH & LH Levels

Test	Result	Reference range
**ACTH (pg/mL)**	**178.0**	**7.2-63.0**
**Baseline 17-OH progesterone (ng/dL)**	**444**	**10-300**
**60-min 17-OH progesterone (ng/dL)**	**837**	**<1000**
Baseline cortisol (mcg/dL)	24.1	8-25
Cortisol after 1mg dexamethasone suppression test (mcg/dL)	0.5	8-24
FSH (mU/mL)	4.1	N/A
LH (mU/mL)	10.0	N/A

Abbreviations: OH = hydroxy; ACTH = adrenocorticotropic hormone; FSH = Follicular Stimulating Hormone; LH = Leutinizing Hormone; N/A = Not Available (due to unknown phase of ovulation)

ACTH stimulation labs bolded.

Genetic testing was performed with CAH gene sequencing and a copy number variation detection panel via Next Gen Sequencing with PGxome exome capture probes to analyze *CYP11A1*, *CYP11B1*, *CYP17A1*, *CYP21A2*, *HSD3B2*, *POR*, and *STAR*, and Sanger sequencing polymerase chain reaction to amplify targeted regions. This revealed a heterogeneous pathogenic *CYP21A2* variant (c.332_339 del; p.Gly111Valfs∗21) and a heterogeneous, previously undescribed variant of unknown significance (VUS) in *CYP11B1* (c.1123C>T; p.Leu375Phe).

The patient was referred to genetic counseling for further discussion of risks to future children of CAH. She was offered oral contraceptives in an attempt to lower her testosterone level until she decided to attempt pregnancy. She was maintained on cabergoline 0.25 mg biweekly but lost to follow-up during the COVID-19 pandemic.

## Discussion

**Fig. 1:**
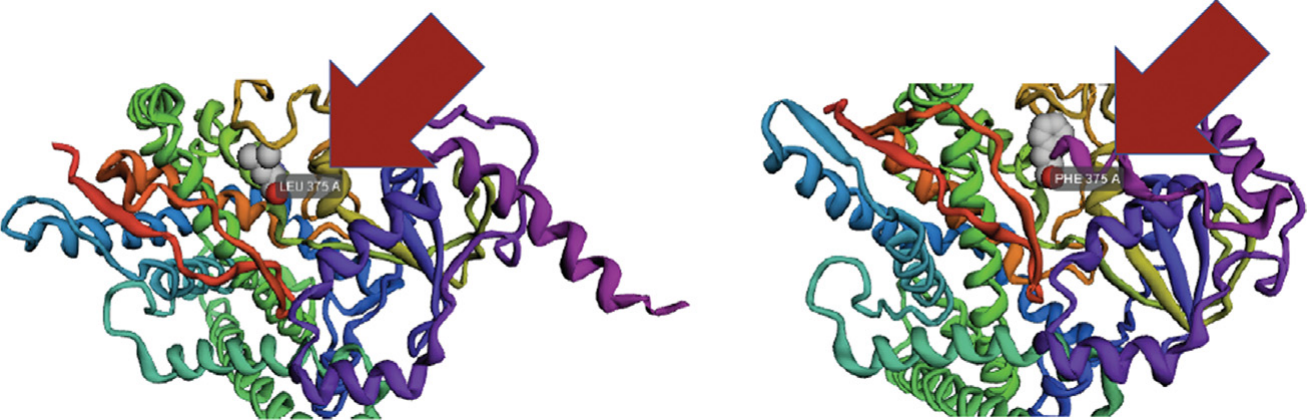
CYP11B1 Wild type and mutant. Arrows indicate the implicated amino acid. Robetta protein structure prediction software demonstrating CYP11B1 wild type (left) and mutant with VUS (p.Leu375Phe) (right).

Our patient presented with symptoms including irregular periods and shaving, both of which were bothersome for the patient. She was treated for hyperprolactinemia without resolution of her symptoms. Additionally, PCOS was considered as an explanation for her presentation; however, she did not fully meet the diagnosis criteria.^11^ In evaluating for NCCAH, she was found to have an elevated 17-hydroxyprogesterone level at baseline prior to adrenocorticotrophic hormone stimulation, although the stimulated 17-hydroxyprogesterone level fell slightly below 1000 ng/dL. Between 1000 ng/dL and 1500 ng/dL is the range considered to be inconclusive.^12^ Given the continued diagnostic uncertainty, genetic testing was performed, and our patient was found to be heterozygous for a known pathogenic *CYP21A2* variant, a G110Efs mutation, c.332_339del, which is predicted to cause a frameshift mutation resulting in premature protein termination per the genetic sequencing laboratory report provided by Prevention Genetics of Marshfield, Wisconsin. Additionally, she was found to be heterozygous in the *CYP11B1* gene for a sequence variant designated c.1123C>T, which is predicted to result in the amino acid substitution pLeu375Phe; this was reported as a VUS. The majority of cases of CAH due to 11β-hydroxylase deficiency present with the previously described classic CAH constellation of findings.^13^ Less commonly, this mutation can lead to NCCAH, primarily when the pathogenic mutation is found outside of exon 8. In another 2018 case study and literature review, Wang et al^14^ described that most mutations in exon 8 result in classic CAH symptoms due to severe disruption of enzymatic structure and activity. In contrast, they reported that most mutations in exon 3 cause more minimal alterations in enzyme structure and, thus, lead to the milder nonclassical presentation. In our case, the mutation was located in exon 7, a location associated with both nonclassic and classic symptoms.^13^^,^^14^ Furthermore, our team used Robetta protein structure prediction software to simulate the mutant *CYP11B1* protein on the basis of our described VUS. The predicted protein structure for wild type and mutant protein is shown in Figure 1. The mutated Phe residue was simulated to be situated near the “roof” of the enzymatic active site and could potentially affect residue binding at this location. In addition, in vitro functional assessment to recreate the mutation and to observe enzymatic changes would be a further method of characterizing her VUS.^15^

Phenotypes of *CYP11B1* deficiencies can be milder and can be similar to nonclassic 21-hydroxylase deficiency.^16^ These milder forms are differentiated, in part, by less virilization and more normotension, as seen in our patient. At least 100 mutations of *CYP11B1* have been found to cause CAH because of 11β-hydroxylase deficiency.^17^ Moreover, a growing number of case reports of novel mutations of *CYP11B1* are adding to the understanding of the genetic variations of this condition.^14^^,^^16^, ^17^, ^18^, ^19^

With respect to combined 21- and 11β-hydroxylase deficiency, a 1985 report described this phenomenon in 5 people among 3 different families.^8^ Two of the individuals in that case report were females whose symptoms included acne and, similar to our patient, hirsutism and menstrual abnormalities. As for the other 3 individuals, 2 were asymptomatic and 1 was a virilized XX female raised as a male. All 5 patients had elevated androgen levels. Our patient’s case differs because she clinically presented with NCCAH. Our patient seems to show that mutations in 2 different genes can potentially present phenotypically as NCCAH. This could enrich earlier conclusions in the literature that broader genetic analysis beyond *CYP21A2* deletions is needed to identify the genotypes of those with CAH due to the diversity of genetic mutations.^20^ Had our patient not had a supportive physical examination and biochemical findings suggestive of NCCAH, her genetic results could have been interpreted to suggest that she is simply a carrier for the 2 genes discussed given the heterozygosity. However, given our findings, it is both intriguing and reasonable to at least consider a digenic cause with heterozygous variants in *CYP21A2* and *CYP11B1* causing her NCCAH. In vitro testing of the VUS and further biochemical testing including 11-deoxycorticosterone, 11-deoxycortisol, or progesterone levels may further indicate whether either the mutation in 21-hydroxylase or 11β-hydroxylase is causing an increase in upstream substrates. This can help indicate whether there is a biochemical significance that we could extrapolate as possibly contributing to the clinical presentation. These tests could reveal a less disease–causing mutation in *CYP11B1* given that our patient was normotensive and without hyponatremia or hyperkalemia. In addition, the VUS in *CYP11B1* should be cataloged and hopefully examined in the context of other patients if found elsewhere, and our patient’s genetic variation, in a way not previously described in case reports or other literature, perhaps contributes to the relative commonness of NCCAH.

## Conclusion

This case demonstrates a patient with physical examination, biochemical, and genetic findings suggestive of NCCAH. Her case suggests that dual heterozygous mutations in separate genes can be seen in NCCAH, which may further help to identify the genotypes of those with NCCAH. We further conclude that our patient’s case ought to remind clinicians to consider NCCAH as a diagnosis, particularly in young women whose hyperandrogenism or menstrual irregularities cannot be explained by PCOS or hyperprolactinemia when the patient is not responding to standard medical treatment.

## Disclosure

The authors have no multiplicity of interest to disclose.
